# Femoral Pseudoaneurysms Requiring Surgical Treatment

**DOI:** 10.5812/kowsar.22517464.3186

**Published:** 2012-01-15

**Authors:** Hannu Savolainen, Iris Baumgartner, Juerg Schmidli, George Heller, Do-Dai Do, Torsten Willenberg

**Affiliations:** 1 Academic Department of Surgery, Queen Elizabeth Hospital, Bridgetown, Barbados; 2 Swiss Cardiovascular Center, University Hospital, 3010 Berne, Switzerland

**Keywords:** Angiography, Catheters, Indwelling, Femoral Artery, Endovascular

## Abstract

**Background:**

Despite use of arterial closure devices (APCDs) and thrombin injection , surgery is needed at times to repair femoral pseudoaneurysms (FPA) in patients undergoing endovascular interventions. We analysed the indications and results of surgical repair in a tertiary referral center performing more than 6.000 angiographies and/or interventions annually.

**Objectives:**

The aim of this retrospective observational study was to identify local and clinical factors related to the need of surgical repair.

**Patients and Methods:**

In this retrospective study, 122 (0.06%) FPAs treated among 21060 patients over a period of five years were assessed. Patient characteristics and therapeutic procedures were analyzed through hospital records.

**Results:**

There were 15.163 (72%) coronary and 5.897 (27%) peripheral interventions, respectively. In 89 (73%) patients, FPA was successfully treated by ultrasound guided compression (USGC) alone.Thirty-three (28%) patients underwent open surgical repair. Indication for operative treatment was hemodynamic instability in 9 (7%) patients, rapidly expanding haematoma unsuitable for USGC or after unsuccessful USGC in 23 (19%). One (0.8%) patient had an arterio-venous fistula. Intraoperative findings suggest that atypical endovascular access (e.g. deep femoral artery, lateral or medial puncture) and multiple puncture sites and/or laceration of the vessel wall were related to the need for surgery in 22 (67%) cases. Most patients had active antithrombotic therapy. Gender or the nature of procedure (diagnostic vs. intervention) did not increase risk for open repair. One (0.8%) patient died. No amputations were performed. Mean hospital stay of patients undergoing open surgical repair was 11 (range 4-36) days.

**Conclusions:**

Technical puncture problems were identified in 2/3 of patients requiring open surgery.

## 1. Background

Along with major complications, such as arterial dissection, occlusion or arterial spasm, FPAs are a rare but serious complication after diagnostic or interventional angiography (1-3). After removal of the catheter sheath, manual compression has previously been used to achieve haemostasis. Recently, manual compression has been largely replaced by prophylactic use of APCDs in many centers. However, in a meta-analysis of 30 randomized trials with more than 5000 patients, concern was raised on the safety of the APCDs, which may actually have a higher incidence of haematoma and pseudoaneurysm than manual compression (4). Several risk factors for vascular complications have been identified, such as female gender, age over 70 years, bleeding disorders, use of thienopyridines, use of glycoprotein IIb/IIIa receptor inhibitors and heparin (5-7). For management of pseudoaneurysm, various techniques have been described. Manual compression is simple but time consuming. Conservative treatment is feasible for small pseudoaneurysms (diameter < 2 cm) (3). USGC (8) and thrombin injection (9) are widely used as well. Nevertheless, a small number of patients will still need open surgical repair (9).

## 2. Objectives

The aim of this retrospective observational study was to identify local and clinical factors related to the need for surgical repair.

## 3. Patients and Methods

21060 patients underwent diagnostic or interventional angiography via femoral arterial access at the Swiss Cardiovascular Center, University Hospital in Berne during a period of five years. USGC was performed by an experienced physician (trained angiologist) for up to 40 minutes using the Acuson 128XP (Mountainview, CA, USA) ultrasound scanning equipment with a 5-MHZ B-mode real time linear array transducer. In 31 (34%) patients further compression after successful ultrasound guided compression was applied using a Femostop device (Radi Medical Systems, Uppsala, Sweden). Statistical analysis was performed using SPSS version? SPSS Inc., Chicago, Ill. software. Chi-square test was used to estimate the role of gender, nature of intervention (diagnostic vs. interventional angiography).

## 4. Results

There were 15.163 (72%) coronary and 5.897 (28%) peripheral interventions. Of them, 122 (0.06%) were treated for subsequent FPA, either by USGC in 89 (73%) patients or by open surgery in 33 (27%). Mean age was 71 years (range 41-91). Patient characteristics are shown in Table 1. Of all the interventions leading to development of a pseudoaneurysm, 78 (64%) were diagnostic or interventional coronary arteriographies. Peripheral diagnostic or interventional arteriographies had been performed in 44 (36%) patients. Surgical repair was performed in 33 patients (27%). In nine (7%) patients, the indication for surgery was a haemodynamically relevant bleeding or shock with a rapidly expanding haematoma. In 23 (19%) patients, there was risk of skin necrosis due to pressure in the haematoma. In one (0.8%) patient surgery was indicated due to an associated arterio-venous fistula.

**Table 1.** Demographic Characteristics on 122 Patients with Iatrogenic Femoral Pseudoaneurysm. Obesity defined as Body Mass Index > 30.

Characteristics	Patients (%)
Arterial hypertension	81
Hyperlipidemia	46
Diabetes mellitus	37
Smoking history	36
Obesity	27

**Figure 1.**
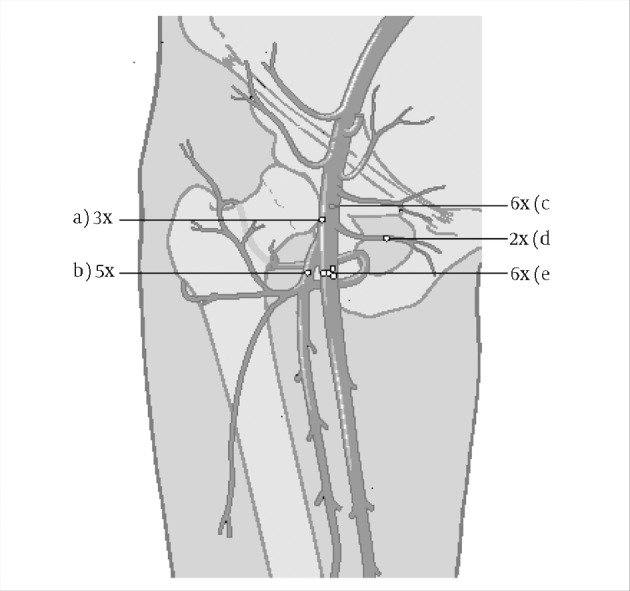
Scheme on intraoperative findings in 22 patients requiring surgical repair due to laceration at the puncture Site. A) Lateral laceration of common femoral artery B) Laceration at profound femoral artery C) Medial or ventral laceration of common femoral artery D) Laceration of a side branch E) More than one laceration

Patient gender or the nature of the procedure (diagnostic vs. interventional) were not statistically significant. Preoperatively, 20 patients (61%) had been anticoagulated (heparin or coumadin). Five (15%) had both clopidogrel and acetosalicylic acid. One patient (0.8%) had undergone thrombolytic therapy for a cerebrovascular insult. One patient (0.8%) had no antithrombotic therapy. In six (18%) cases, there was no reliable information on current antithrombotic therapy.Of the operations, 30 (91%) were performed under general anesthesia, 3 (9%) under spinal or epidural analgesia. Seven (21%) pseudoaneurysms were managed with a staged procedure using vacuum-assisted closure (VAC, KCI Inc., San Antonio, Tx, USA). Five (15%) patients developed a wound infection requiring further surgical therapy before secondary closure. Intraoperative findings suggested a technical puncture problem in 22 (67%) patients (Figure 1). A lateral laceration of the common or superficial femoral artery (CFA) was seen in three (9%) cases. A medial or ventral laceration CFA was described in six (18%) cases. A laceration of a side branch of the superficial femoral artery was seen in two (6%) patients. In five (15%) cases, the deep femoral artery had been accessed. Multiple bleeding puncture sites were seen in six (18%) patients. Mean hospital stay after open repair was 11 days.

## 5. Discussion

A pulsatile hematoma, pseudoaneurysm, may be the result of any vascular intervention. It may lead to serious consequences, such as major amputation or even death (9). FPA occurs in 0.1-0.2% after arteriographies (1-3). In our series of more than 21.000 arterial interventions, the occurrence was 0.06%. Various APCDs have recently been introduced to prevent haematoma formation. They include collagen plugs with or without an anchor from inside the artery (10, 11), balloon-positioning catheters combined with bovine collagen and thrombin (12, 13). External mechanical compression devices are available as well (14). Although time to hemostasis and duration of bed rest are shorter with APCDs, most studies have been heterogeneous. A meta-analysis of 30 randomized trials showed only marginal evidence that APCDs are more effective. According to the analysis, they may even increase the risk of FPA (4). Dissections and embolizations have been seen in connection with their use as well (5). There are few data regarding indications for surgical repair of FPAs. In our series, USGC was successful in 73%. Still, one third were operated on emergently, mainly due to progressive local findings or haemodynamic instability. Operative exposure of the artery suggested problems at the puncture site in two thirds, either laceration or more than one bleeding puncture site. In our experience, manual compression is not feasible in extremely painful FPAs - independently of morphology-associated with a rapidly expanding haematoma, as pressure in the haematoma may lead to skin necrosis. In case of haemodynamic instability, operative treatment is expeditious and definitive.

Several antithrombotic regimes have been described as risk factor FPA, such as heparin, thienopyridines or glycoprotein receptor inhibitors (5-7). Although our data were not complete with regard to current coagulation status, at least two thirds were actively treated with coumadine or platelet inhibitors. Mortality rates of up to 5% have been reported in association with iatrogenic arterial injuries (15). Major amputations have been performed in up to 2, 3 %(16). In our series, there were no major amputations, but one patient (0.8%) died due to myocardial infarction after repair. The initial indication for therapy was acute myocardial infarction. Local wound complications requiring further surgical therapy were seen in 15% of patients, leading to prolonged hospitalisation. In these patients, the initial haematoma was very large. A surgical incision in such an area after skin puncture for angiography naturally favors infection.

Preventing pseudoaneurysm formation is a strategy. Our study shows that manual compression alone is effective in 99% of patients undergoing arterial access for diagnostic purposes or endovascular interventions. Minimizing the risk of FPA requires careful puncture technique. Aggressive antithrombotic therapy increases the risk of FPA. The main limitation of our study is the relatively small number of patients and its retrospective nature. The use of glycoprotein IIb/IIIa inhibitors was not always recorded. In conclusion, FPA requiring surgery is a rare but serious complication after diagnostic or interventional angiography. Two thirds of FPAs requiring surgery may be the result of poor puncture technique and inadvertent lesions of the arterial wall. Endovascular access should be planned and executed carefully. In experienced hands, open repair is straightforward.
